# Modifiable lifestyle factors for dementia risk in an online cohort assessed by the MoCA Cognitive Health Assessment Index (MoCA-CHAI)

**DOI:** 10.1016/j.tjpad.2026.100546

**Published:** 2026-03-27

**Authors:** Laura Klaming, Hans-Aloys Wischmann, Murray Gillies, Ziad Nasreddine

**Affiliations:** a MoCA Test Inc., 4896 Blvd Taschereau, Greenfield Park, Quebec J4V 2J2, Canada; b Institute of Public Health, Charité – Universitätsmedizin Berlin, Berlin, Germany

**Keywords:** Cognitive health, Lifestyle, Dementia risk, Modifiable risk factors, Prevalence, Risk assessment, Surveys and questionnaires, Real-world data

## Abstract

**Background:**

The growing prevalence of dementia highlights the need for a risk assessment tool that is accessible, facilitates the identification of at-risk individuals, and provides evidence-based guidance on how to reduce dementia risk.

**Objectives:**

We developed, deployed, and evaluated the MoCA-CHAI, a self-administered, online dementia risk assessment for the general public. We provide a brief overview of its development, the self-enrolled population that has completed it, and a preliminary evaluation of its predictive performance.

**Method:**

Drawing on the 2024 *Lancet* report, we developed the MoCA-CHAI translating the 14 identified risk factors into a questionnaire. We used the MoCA XpressO as a measure of cognitive impairment and a proxy measure for the probability of having or developing dementia.

**Findings:**

The MoCA-CHAI was completed by 3886 people. Based on their XpressO score, 11.3% showed a high probability of cognitive impairment. Using a logistic regression analysis, we found that each 1-point increase in MoCA-CHAI score decreases the odds of having cognitive impairment by 1%. Physical inactivity and exposure to air pollution are the most prevalent risk factors across the lifespan. Depression is more prevalent in young adults, while high cholesterol, hypertension, diabetes, and excessive alcohol consumption are more prevalent in middle-aged and older adults.

**Conclusions:**

These findings demonstrate that the MoCA-CHAI provides insight into modifiable lifestyle factors and dementia risk. Differences in prevalences of risk factors indicate that prevention strategies need to be tailored to age-specific risk profiles. The MoCA-CHAI may help identify at-risk individuals who could benefit from targeted prevention and monitoring.

## 1 Introduction

Given the projected trends in population aging and growth, the number of people living with dementia worldwide is projected to almost triple from an estimated 57 million in 2019 to an estimated 153 million in 2050 [1]. This projected growth underscores the need for public health policy to prioritise the assessment of risk factor prevalence and the reduction of modifiable risk factors for cognitive impairment and dementia at the population level.

Research has consistently shown that several lifestyle and medical factors are predictors for the development of cognitive impairment and dementia later in life, which has led to a growing interest in risk assessment and prevention approaches. Over the past 20 years, several dementia risk indices have been developed, e.g. the Lifestyle for Brain Health (LIBRA; [2]), the Cardiovascular Risk Factors, Aging, and Incidence of Dementia score (CAIDE; [3]), and the Late-life Dementia Risk Index [4]. These risk indices are typically derived from single cohort studies, are often based on a limited number of risk factors, and sometimes include non-modifiable risk factors such as age, gender, apolipoprotein E genotype and neuroimaging markers such as white matter disease and atrophy [4,3]. Basing a risk assessment on a single cohort study is problematic because the findings may not be generalisable beyond its study population. Risk factors extracted from neuroimaging and genotyping are costly, time consuming and often not easily available to the general public, and provide insights into non-modifiable rather than modifiable risks. In addition, most risk indices are not accessible to the public limiting their practical value for early risk stratification and timely prevention. There is a need for a concise, yet comprehensive, easily accessible dementia risk assessment tool that is based on modifiable risk factors and that not only provides users with an indication of their current dementia risk, but moreover provides useful and evidence-based information about actions that can decrease their dementia risk.

Based on previous research, the 2024 report of the *Lancet* standing Commission describes 14 modifiable risk factors for dementia and provides the risk ratios and population attributable fractions for each of the risk factors based on their prevalence and relation with dementia [5]. The 14 modifiable risk factors are: low educational attainment, uncorrected hearing loss, high cholesterol, depression, traumatic brain injury, physical inactivity, smoking, diabetes, hypertension, obesity, excessive alcohol consumption, social isolation, air pollution, and untreated vision loss. For some of these factors, there are differential effects across the lifespan. While reducing or avoiding risk factors as early as possible in life is beneficial, most risk factors require attention across the entire lifespan. Although many of the risk factors are modifiable at the individual level, there is also a need for policy interventions at the population level. In theory, the prevalence of dementia could be nearly cut in half if these 14 modifiable risk factors could be fully eliminated [5].

While the *Lancet* study quantifies the risk, there is no easy way for the interested public to assess their dementia risk. To address this unmet need, we developed the MoCA-Cognitive Health Assessment Index (MoCA-CHAI) ,^1^ based on the 14 risk factors identified in the 2024 *Lancet* report, as a web-based tool that can be used by individuals to assess their dementia risk.

## 2 Objective

The objective of the present study is to provide a brief overview of the development of the MoCA-CHAI, to describe the self-enrolled population that has completed the MoCA-CHAI in the first six months after its launch, and to provide a preliminary evaluation of its risk prediction performance using the MoCA XpressO as a measure for current cognitive status and as a proxy measure for the probability of having or developing dementia.

## 3 Methods

### 3.1 Development of the MoCA-CHAI

Drawing on the 2024 report of the *Lancet* standing commission, we developed the MoCA-CHAI translating the 14 identified risk factors into a questionnaire. For two of the risk factors (physical inactivity, excessive alcohol consumption), we included two questions which were subsequently combined into one score per question. Five of the questions (uncorrected hearing loss, high cholesterol, diabetes, hypertension, untreated vision loss) also included follow-up questions if the question was answered affirmatively. For instance, if the individual indicated that he or she had hearing difficulties, the follow-up question asked whether they could hear adequately with the use of a hearing aid or other device. For uncorrected hearing loss and untreated vision loss, answers to these follow-up questions were used in the score, i.e. if the individual indicated that he or she had hearing difficulties, but could hear adequately with the use of a hearing aid or other device, uncorrected hearing loss was not considered a risk factor. Answers to the follow-up questions for high cholesterol, diabetes, and hypertension did not factor into the MoCA-CHAI score, so that a diagnosis of high cholesterol, diabetes, or hypertension was considered a risk factor, even if the individual indicated in the follow-up question that they take medication to treat it.

Besides questions based on the 14 risk factors identified in the 2024 *Lancet* report, we also included an additional 10 questions about modifiable lifestyle factors that can indirectly affect dementia risk. These factors include sleep [6,7], diet [8,9], and cognitive and social activity such as reading and socialising with friends and family [10,11]. Studies often, but inconsistently found associations between these factors and cognitive impairment and dementia, partially because it is unclear whether some factors are a cause or a consequence of cognitive decline. The answers to these additional questions did therefore not factor into the MoCA-CHAI score, but they were included in the questionnaire since they may indirectly affect dementia risk and offer users guidance about how to adapt behaviours or lifestyle.

The different risk factors included in the MoCA-CHAI were grouped into five categories: physical health, emotional well-being, diet, exercise, and cognitive and social engagement. Users receive a total brain health score that ranges from 0 to 100, with higher scores indicating fewer risks and thus better protection against developing dementia. In addition, sub-scores are provided for each of the five categories based on both the 14 identified risk factors and the additional factors that may indirectly affect dementia risk to provide users with more detailed information about the areas in which they could modify their lifestyle. After completing the MoCA-CHAI, individuals receive personalised recommendations about how they can adjust their lifestyle to reduce their individual risk of dementia.

### 3.2 The MoCA-CHAI score

Based on the risk ratio (RR) of each of the 14 risk factors as reported in the 2024 *Lancet* report and obtained from meta-analyses across multiple studies and cohorts, we developed an algorithm to provide a total brain health score, the MoCA-CHAI score. Assuming an additive risk model, we converted the RR for each risk (r) into a scaled risk difference (RD), i.e. a weight or point score associated with this risk, and summed the resulting weights across risks to which a participant was exposed. Whenever data was available, the weight for each risk factor furthermore reflected different impacts of risk factors early in life, in midlife, and late in life by varying the weight based on the individual's age.

Specifically, for each of the 14 risk factors r, a weight was computed from the RR:

(1) $$Weight_{r}=100*\left(RR_{r}-1\right)$$

Then, to create a risk score for a person p, weights were summed for the risks e(p) the person is or was exposed to:

(2) $$Risk_{p}=\sum Weight_{e\left(p\right)}$$

The maximum possible risk was calculated by adding the maximum weights of all 14 risk factors:

(3) $$MaxRisk=\sum MaxWeight_{r}$$

Subsequently, a total MoCA-CHAI score was calculated by dividing the risk score for the person (Risk_p_) by the maximum possible risk (MaxRisk). The MoCA-CHAI score was scaled to range between 0 (maximum risk) and 100 (maximum protection):

(4) $$CHAI=100*\left(1-\left(Risk_{p}/MaxRisk\right)\right)$$

Table 1 provides an overview of the RRs for dementia from the 2024 *Lancet* report and its appendix and supplementary materials, together with the points for each risk factor taking into account different exposure levels within some of the risk factors and the age of the respondent to account for differential effects of the risk factor in early life, midlife, and late life whenever available.

**Table 1:** Risk factors and risk ratios (RR) from the Lancet report and points assigned for the MoCA-CHAI score.

Risk factor	Risk ratio (RR) (95 % CI)	Points	
Low educational attainment	1.6 (1.3–2.0)	59	primary school
		33	≥ some college, no degree
		0	≥ bachelor's degree
Uncorrected hearing loss	1.4 (1.0–1.9)	37	untreated hearing impairment
		0	no or treated hearing impairment
High cholesterol	1.3 (1.3 - 1.4)	32	high cholesterol
		0	no high blood cholesterol
Depression	2.2 (1.7–3.0)	125	depression
		0	no depression
Traumatic brain injury	1.7 (1.4–1.9)	72	traumatic brain injury
		0	no traumatic brain injury
Physical inactivity	1.2 (1.2–1.3)	12	never aerobic exercises
		8	strength aerobic 1 time/week
		4	strength aerobic 2 times/week
		0	strength aerobic 3+ times/week
		12	never strength exercises
		8	strength exercises 1 time/week
		4	strength exercises 2 times/week
		0	strength exercises 3+ times/week
Smoking	1.3 (1.2–1.4)	30	currently smoking
		0	not (currently) smoking
Diabetes	1.7 (1.6–1.8)	73	diabetes
		0	no diabetes
Hypertension	1.2 (1.1–1.4)	20	hypertension
		0	no hypertension
Obesity*	1.3 (1.0–1.7)	64	BMI <18.5 and age <65 years
		56	BMI >30 and age<65 years
		50	BMI <18.5 and age ≥65 years
		39	BMI 25–30 and age <65 years
		25	BMI 18.5–25
		6	BMI 25–30 and age ≥65 years
		0	BMI >30 and age ≥65 years
Excessive alcohol consumption⁎⁎	1.2 (1.0–1.5)	21	drinking ≥4 drinks, 4+ times a week
			drinking ≥6 drinks, 2–3 times a week
		15	1–3 drinks, 4+ times a week
			4–5 drinks, 2–3 times a week
		0	1–3 drinks, 2–3 times a week
			drinking ≤2–4 times per month
Social isolation	1.6 (1.3–1.8)	57	living alone
		0	living with someone
Air pollution	1.1 (1.1–1.1)	12	above average air pollution
		6	average air pollution
		0	below average air pollution
Untreated vision loss	1.5 (1.4–1.6)	47	uncorrected vision loss
		0	no or corrected vision loss

The risk ratios (RR) are from Livingston et al. [5].

⁎ For BMI, point scores were shifted by 25 points so that the lowest point score was always 0.

⁎⁎ 1 drink is equal to 1.5 units [18]. 21 units (equal to 14 drinks) and 14 units (equal to 9 drinks) per week were used as thresholds for heavy drinking and for moderate drinking, respectively.

### 3.3 Study

The MoCA-CHAI was evaluated in a large population-based observational study using real-world data. We collected data in a self-enrolled online cohort between February 2025 and July 2025. The study was conducted in compliance with the declaration of Helsinki. Participants provided electronic informed consent for use of their anonymised data for research purposes and completed the MoCA-CHAI and the MoCA XpressO online. The XpressO is a brief online self-assessment tool that has been developed by MoCA Test Inc. as a pre-screener for cognitive impairment, with details described previously [12]. It generates a probability score that predicts whether a participant would score below 25 on the MoCA test indicating cognitive impairment. XpressO scores can range between 0 and 100, with scores <42 predicting a score of ≤24 on the MoCA with a specificity of 90 % [12]. A score of <42 on the XpressO was used in this study as a measure for cognitive impairment and as a proxy measure for the probability of having or developing dementia. While a longitudinal cohort study design would provide direct evidence of dementia risk over longer periods of time, a proxy measure was used for this initial study to assess the relation between lifestyle factors and dementia risk. The XpressO was administered remotely and unsupervised via an application installed on a mobile device or in a web browser.

### 3.4 Statistical analyses

To examine whether MoCA-CHAI scores predict dementia risk, defined as having an XpressO score <42, a binary logistic regression analysis was conducted, adjusting for age, gender, and the interaction between age and gender. Logistic regression coefficients (β) represent changes in the log-odds for having cognitive impairment for a one-unit increase in each predictor. Odds ratios (ORs) were calculated by exponentiating the coefficients (*e*^β^). Confidence intervals (95 %-CIs) were calculated by exponentiating the lower and upper limits derived from the standard error (SE) of each coefficient (*e*^β±1.96*SE^).

To assess and overcome limitations of assumed linearity in the logistic regression, a sensitivity analysis using Generalized Additive Models (GAMs) was performed using smooth functions for age, by sex. Marginal risk ratios were calculated for the main regression model and for the sensitivity analyses.

Statistical analyses and visualizations were conducted using Python (including libraries: Pandas 2.0.3, Numpy 1.25.2, Statsmodels 0.14.3, Matplotlib 3.7.2, Seaborn 0.13.2) and R (4.5.1).

## 4 Results

The total sample of *N* = 3886 individuals is predominantly female (60.4 %) and highly educated (71.4 % completed tertiary education) with a mean age of 54 years (SD=16 years). The mean MoCA-CHAI score is 77.6 (SD=13.4) and the mean MoCA XpressO score is 77.3 (SD=23.4). Slightly more than a tenth of the sample, *N* = 440 (11.3 %), scored below the <42 threshold of the MoCA XpressO and are therefore considered to have a high probability of suffering from cognitive impairment. The mean age of this subgroup is 61 (SD=15.9) years. Demographic information as well as MoCA-CHAI and XpressO scores are summarised in Table 2.

**Table 2:** Demographic information, MoCA-CHAI and MoCA XpressO scores.

		N ( %)	Mean (SD)
			Median (Min, Max)
Gender	Female	2347 (60.4 %)	
	Male	1539 (39.6 %)	
Age (years)			53.9 (15.8)
			56 (18, 94)
Education level	Tertiary	2774 (71.4 %)	
	Post-secondary non-tertiary	774 (19.9 %)	
	Upper secondary	272 (7.0 %)	
	Lower secondary	57 (1.5 %)	
	Primary	9 (0.2 %)	
Country	United States	1540 (39.6 %)	
	Canada	400 (10.3 %)	
	Other	118 (3.1 %)	
	Missing	1828 (47 %)	
MoCA-CHAI			77.6 (13.4)
			80.8 (28, 100)
MoCA XpressO			77.3 (23.4)
			86.3 (5, 100)

**Fig. 1:**
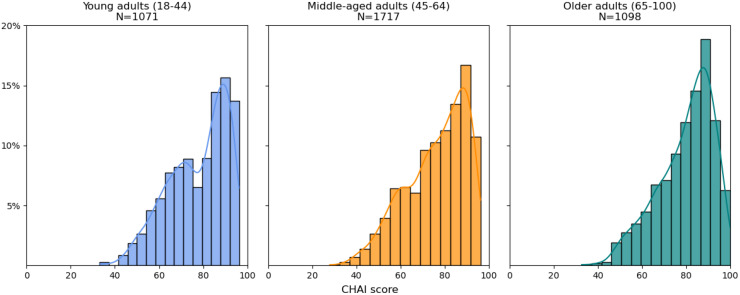
Distribution of CHAI score stratified by age group.

Fig. 1 displays the distribution of the CHAI scores in young, middle-aged, and older adults. The mean CHAI score in young adults is 77.6 (SD=13.3) compared to 76.2 (SD=13.8) in middle-aged adults and 79.8 (SD=12.5) in older adults, reflecting a slightly higher prevalence of risk factors in young and middle-aged adults, and showing a bimodal structure in young and middle-aged persons.

**Fig. 2:**
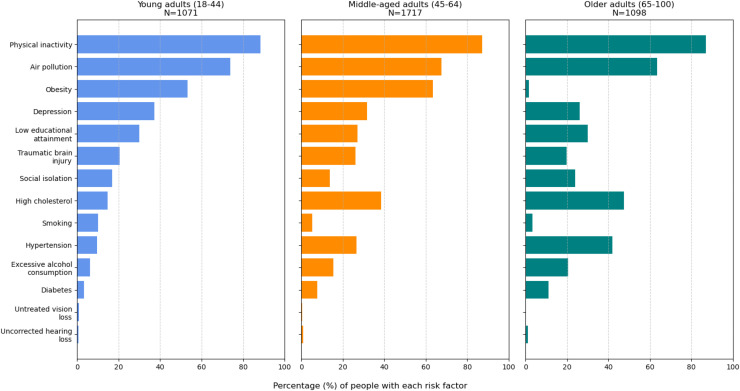
Prevalence of each risk factor (in %) stratified by age group.

Participants reported a wide range of lifestyle and health-related risk factors for dementia with the majority reporting between three and five risk factors, where a person was counted as exposed to a risk whenever they scored more than 0 points for a factor, i.e., an exposure other than the healthiest level. The most prevalent risk factors were physical inactivity (87.4 %), exposure to air pollution (68.1 %), unhealthy BMI for their age (43.1 %), and high cholesterol (34.5 %). Fig. 2 shows the prevalence of each risk factor in young, middle-aged and older adults. While depression is more prevalent in young adults, high cholesterol, hypertension, diabetes and excessive alcohol consumption are more prevalent in middle-aged and older adults.

A logistic regression was conducted to examine whether MoCA-CHAI score, gender, age, and the interaction between gender and age predicted the probability of suffering from cognitive impairment, defined as having an XpressO score <42. The overall model fit is acceptable, Pearson χ²(3880) = 4060, with a Nagelkerke *R^2^* of 0.04, indicating modest explanatory power. As can be seen in Table 3, higher MoCA-CHAI scores are associated with reduced odds of dementia risk (β=-0.011, *SE*=0.004, *p*=.003, OR=0.99). For each 1-point increase in MoCA-CHAI score, the odds of having cognitive impairment decrease by about 1 %, and the marginal risk ratio for a 1-point increase in the MoCA-CHAI score is 0.990 (see Appendix). Older age significantly predicts the odds of being in the cognitive impairment risk group (β=0.028, *SE*=0.005, *p*<.001, OR=1.03). For each additional year of age, the odds increase by about 3 %. There was also a significant interaction between age and gender with a stronger effect of age on cognitive impairment risk in men than in women (β=0.017, *SE*=0.008, *p*<.025, OR=1.02). Gender alone was not a significant predictor (*p*=.212).

**Table 3:** Logistic regression predicting low XpressO scores (<42) indicative of cognitive impairment and probability of having or developing dementia.

Predictor	β	SE	OR [95 %-CI]	p
Male (vs. Female)	-0.579	0.464	0.56 [0.23, 1.39]	0.212
MoCA-CHAI score	-0.011	0.004	0.99 [0.98, 1.00]	0.003
Age	0.028	0.005	1.03 [1.02, 1.04]	<0.001
Age*Male	0.017	0.008	1.02 [1.00, 1.03]	0.025

**Fig. A1:**
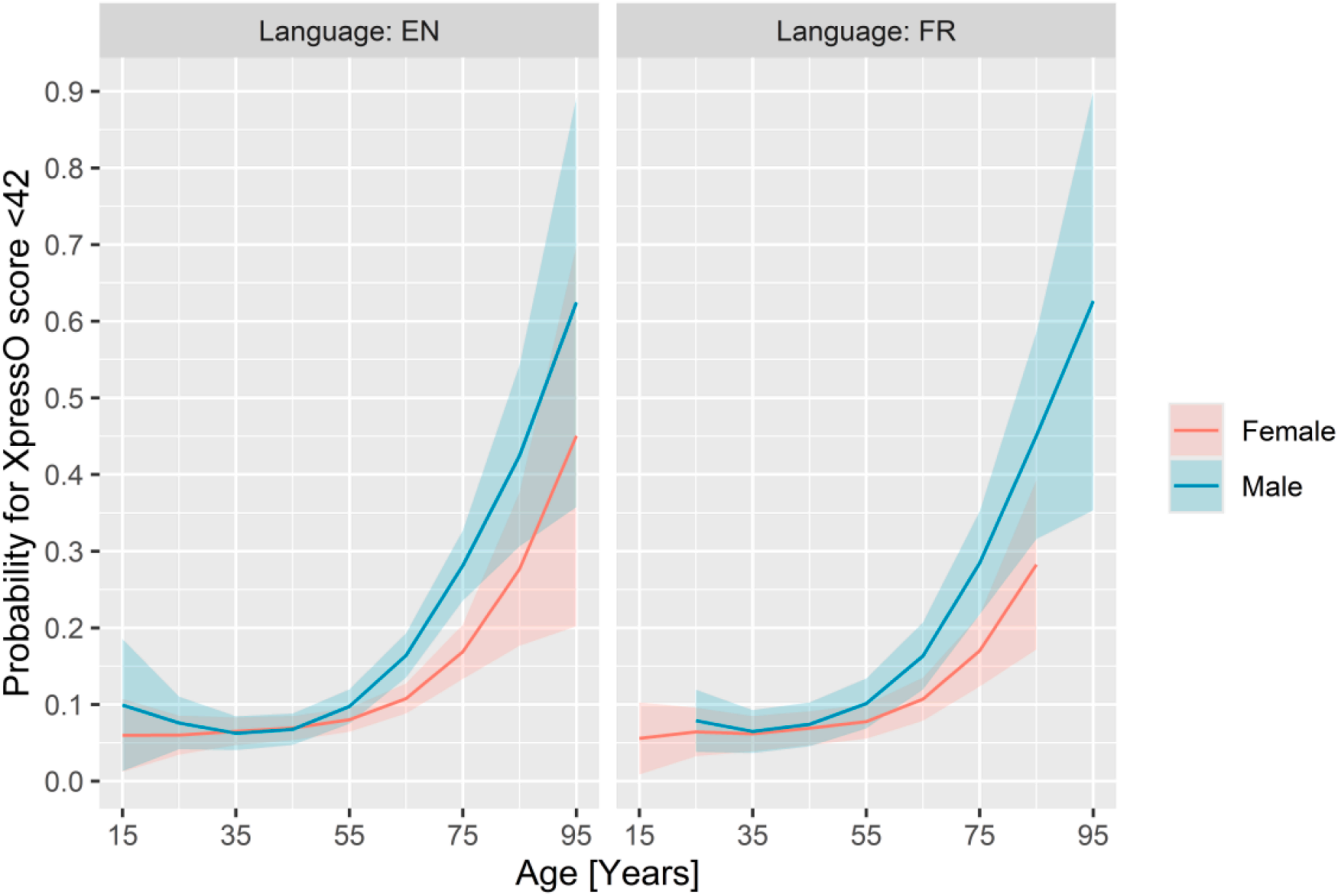
Age dependency for low XpressO scores (<42) indicative of cognitive impairment and probability of having or developing dementia, with 95 %-CI, stratified by gender and language of the CHAI, for the observed mean CHAI score by gender, language, and age group, as predicted by the GAM logistic regression.

These findings are confirmed in the sensitivity analysis (see Appendix Table A1), where the language of the test was added to the potential predictors/confounders for the logistic regression, but was not statistically significant. The smooth age dependencies in the sensitivity analysis using GAMs (see Appendix Table A2 and Fig. A1) also exhibited gender differences, and gender is statistically significant in this model, with higher odds for male persons (OR=1.373, 95 %-CI=[1.098, 1.718], *p*=.006).

## 5 Discussion

There is growing recognition that lifestyle factors are critical for the reduction of dementia risk later in life. The MoCA-CHAI provides a brief, easy-to-use, online lifestyle questionnaire to assess dementia risk, drawing from existing research. The finding that it has been used widely since its launch in January 2025 suggests that it meets a need experienced by a significant part of the population for accessible tools enabling individuals to understand and act on modifiable risk factors for dementia.

We found that 11.3 % of the self-enrolled online cohort scored below the <42 threshold of the MoCA XpressO and therefore have a high probability of suffering from cognitive impairment. The mean age of this subgroup was slightly higher than the mean age of the entire sample. The prevalence of 11.3 % is similar to the prevalence of mild cognitive impairment found in studies including community-dwelling adults over 55 years [13]. Additionally, we found that the majority of our sample had between two and six risk factors with physical inactivity, exposure to air pollution, obesity, and high cholesterol being the most prevalent. The prevalence of some of these risk factors differ from those reported in the 2024 *Lancet* report, likely reflecting differences in populations. As at least 40 % of our sample are from the US where higher rates of lifestyle-related and cardiometabolic risk factors have been documented [17], this may partly explain the elevated prevalence observed regarding physical inactivity and obesity in our sample. Additionally, it is known that self-enrolled study participants are generally more highly educated, healthier, and more interested in learning about their health [14]. The fact that self-enrolled cohorts are generally healthier may explain why the prevalences of cardiometabolic risk factors is lower in our sample than the prevalences reported in the 2024 *Lancet* report.

We also found that the prevalence of risk factors and consequently the average CHAI score differs with age in our study sample, with a slightly higher prevalence of risk factors in young and middle-aged adults. While physical inactivity and exposure to air pollution were the most prevalent risk factors in all age groups, depression was more prevalent in young adults, while high cholesterol, hypertension, diabetes, and excessive alcohol consumption were more prevalent in middle-aged and older adults. This indicates that prevention strategies need to be tailored to age-specific risk profiles, prioritising mental health and lifestyle interventions in young adults and cardiovascular risk management in middle-aged and older adults.

By using the MoCA XpressO as a proxy for the probability of developing dementia, we were able to show that higher MoCA-CHAI scores are protective with each 1-point increase in CHAI score decreasing the odds of being in the low XpressO group by 1 %. This finding suggests that the MoCA-CHAI predicts cognitive impairment risk and that a healthier lifestyle as measured by the MoCA-CHAI decreases the risk of developing dementia later in life. Since age has a large effect on XpressO score, the contribution of age in the regression model was also significant with each 1-year increase in age raising the odds of being in the low XpressO group by 3 %. The effect of age was found to be slightly stronger for men. The finding that age is a robust predictor of cognitive impairment risk is consistent with prior research demonstrating that age is one of the strongest predictors of dementia [15]. The finding that the MoCA-CHAI score predicts cognitive impairment suggests that it captures variations in risk relevant to cognitive health and supports its utility as a risk assessment index for the general population. Further validation in larger and more diverse cohorts is warranted to determine its generalisability.

### 5.1 Strengths and limitations

A major strength of the implemented online MoCA-CHAI risk assessment tool is that it is based on the 2024 *Lancet* report to ensure that it covers relevant risk factors that have consistently been shown to predict dementia risk. The strength of the study is the use of a large online cohort, consisting of individuals who are intrinsically motivated to learn more about their dementia risk and ways to improve their lifestyle to reduce their risk. The digital format of the MoCA-CHAI makes it an efficient, accessible tool to investigate modifiable risk factors for dementia.

The study has a number of important limitations. Since participants self-enrolled, the sample is subject to selection bias. The majority of the sample is highly educated and probably healthier than the general population. Additionally, the sample may overrepresent individuals who are more interested in their health and more motivated to change their lifestyle to decrease their risk of cognitive impairment. This limits generalisability, particularly to populations with lower socioeconomic status, limited digital access, or higher risk of cognitive impairment who may benefit most from an assessment tool like the MoCA-CHAI.

Related to this, risk factor prevalence and sociocultural influences may vary across countries. Since at least half of the study population is from the US and Canada, this limits the generalisibility of the findings. Additional validation in other populations from different countries and cultural backgrounds is therefore needed.

Another limitation concerns some of the risk factors included in the MoCA-CHAI. We have included low educational attainment as a risk factor although it may be argued that education is non-modifiable. We have decided to include it based on the findings of the 2024 *Lancet* report which highlights the benefits of continued learning and cognitive stimulation throughout the lifespan [5]. In addition, the measurement of certain risk factors is inherently inaccurate. For instance, we measure exposure to air pollution with one question while people can be exposed to different levels of air pollution throughout their lives depending on where they live and whether they are exposed to additional air pollution at work. Obesity was measured using BMI. While widely used, BMI does not account for fat distribution, which is more strongly linked to cardiometabolic outcomes, but less straightforward to measure [16]. The way some risk factors are measured therefore carries a degree of unavoidable error which could bias individual risk and prevalence estimates.

An important limitation of this study is the fact that all risk factors and dementia risk were measured at a single point in time rather than in a longitudinal study with risk factors measured at baseline and dementia incidence at a later point in time. The probability of developing dementia was measured using the MoCA XpressO as a proxy measure. This is an important shortcoming because using a proxy measure likely underestimates the prevalence of true cognitive impairment or dementia risk. In addition, the XpressO is not validated as a diagnostic measure for dementia. Longitudinal studies are needed to further validate the MoCA-CHAI.

At present, the MoCA-CHAI does not account for a cumulative effect of risk factors and only partially accounts for the differential effect of risk factors across the lifespan. This limitation reflects the limited availability of data necessary to robustly estimate differential effects across the lifespan. A future version may address this by incorporating weights that reflect the impact of certain combinations of risk factors as well as by incorporating more age-stratified data as it becomes available to allow for more precise estimation of differential effects.

As with any population-based predictive model, risk estimates derived from the MoCA-CHAI reflect aggregated probabilities across groups and do not account for individual-level variability. They are not deterministic, so that outcomes for individuals will vary, even if the mean predictions are correct.

### 5.2 Clinical implications

Even though the MoCA-CHAI has been developed to be used by the general public, it may also be of value to clinicians who need reliable and valid tools to assess dementia risk and to policy makers who need to identify at-risk groups to provide targeted prevention strategies. As such, the MoCA-CHAI may be a valuable tool to be recommended by primary care physicians to their interested patients and for use in routine clinical practice to efficiently identify high-risk individuals who may benefit from more frequent monitoring and early intervention. Gaining insight into modifiable risk factors can provide both the person at risk and their clinicians with a practical starting point for lifestyle interventions.

In addition to its direct clinical implications, the MoCA-CHAI may provide a strategy for enrolling high-risk individuals in clinical trials and intervention studies.

## 6 Conclusion

The MoCA-CHAI expands the scope of cognitive assessments with self-reported indicators of lifestyle and health thereby addressing the need for a multidimensional approach to cognitive health. The large number of individuals who have filled in the MoCA-CHAI since its publication shows that the instrument addresses a public need for a reliable and accessible online assessment of dementia risk and evidence-based guidance on modifiable lifestyle factors to support cognitive health. Future directions include combining the MoCA-CHAI with the MoCA in populations with known MCI and early-stage dementia as well as using the MoCA-CHAI in longitudinal studies to validate its predictive performance.

## Declaration of generative AI and AI-assisted technologies in the writing process

The authors have not used any AI or AI-assisted technologies in the process of writing this manuscript.

## Authorship statement

Laura Klaming led the development of the MoCA-CHAI. All other authors contributed to the development of the MoCA-CHAI. Laura Klaming and Hans-Aloys Wischmann analyzed the data and drafted the manuscript. Murray Gillies and Ziad Nasreddine were involved in critically revising the manuscript.

## Funding

The work and the authors Laura Klaming, Murray Gillies, and Ziad Nasreddine were funded by MoCA Test Inc.

## Data statement

Due to the sensitive nature of the questions asked in this study, survey respondents were assured raw data would remain confidential and would not be shared.

## CRediT authorship contribution statement

**Laura Klaming:** Writing – original draft, Visualization, Methodology, Formal analysis, Conceptualization. **Hans-Aloys Wischmann:** Writing – original draft, Visualization, Formal analysis. **Murray Gillies:** Writing – review & editing, Project administration. **Ziad Nasreddine:** Writing – review & editing, Supervision.

## Declaration of interest

The authors declare that they have no known competing financial interests or personal relationships that could have appeared to influence the work reported in this paper.

## Figures and Tables

**Table A1:** Sensitivity analysis with language of CHAI test, logistic regression predicting low XpressO scores (<42) indicative of cognitive impairment and probability of having or developing dementia.

Predictor	OR [95 %-CI]	p.value
CHAI	0.989 [0.981, 0.996]	0.003
French (vs. English)	0.991 [0.741, 1.309]	0.95
Other (vs. English)	0.708 [0.401, 1.167]	0.20
Male (vs. Female)	0.558 [0.223, 1.377]	0.21
Age	1.028 [1.017, 1.038]	<0.001
Male:Age	1.017 [1.002, 1.032]	0.023

**Table A2:** Sensitivity analysis with language of CHAI test, logistic regression predicting low XpressO scores (<42) indicative of cognitive impairment and probability of having or developing dementia, using generalized additive models (GAMs) with smooth terms for age by gender.

Predictor	OR [95 %-CI]	p.value
CHAI	0.987 [0.979, 0.994]	<0.001
French (vs. English)	1.019 [0.765, 1.358]	0.90
Other (vs. English)	0.719 [0.423, 1.223]	0.22
Male (vs. Female)	1.373 [1.098, 1.718]	0.006

**Table A3:** Marginal risk ratios (RRs) for low XpressO scores (<42) indicative of cognitive impairment and probability of having or developing dementia, for a 1-point increase in the MoCA-CHAI score, across the various logistic regression models.

Regression model	Marginal RR [95 %-CI]	p.value
Main model (glm)	0.990 [0.984, 0.997]	<0.001
Sensitivity model (glm)	0.990 [0.984, 0.997]	<0.001
Sensitivity model (gam)	0.989 [0.983, 0.995]	<0.001

**Fig. A2:**
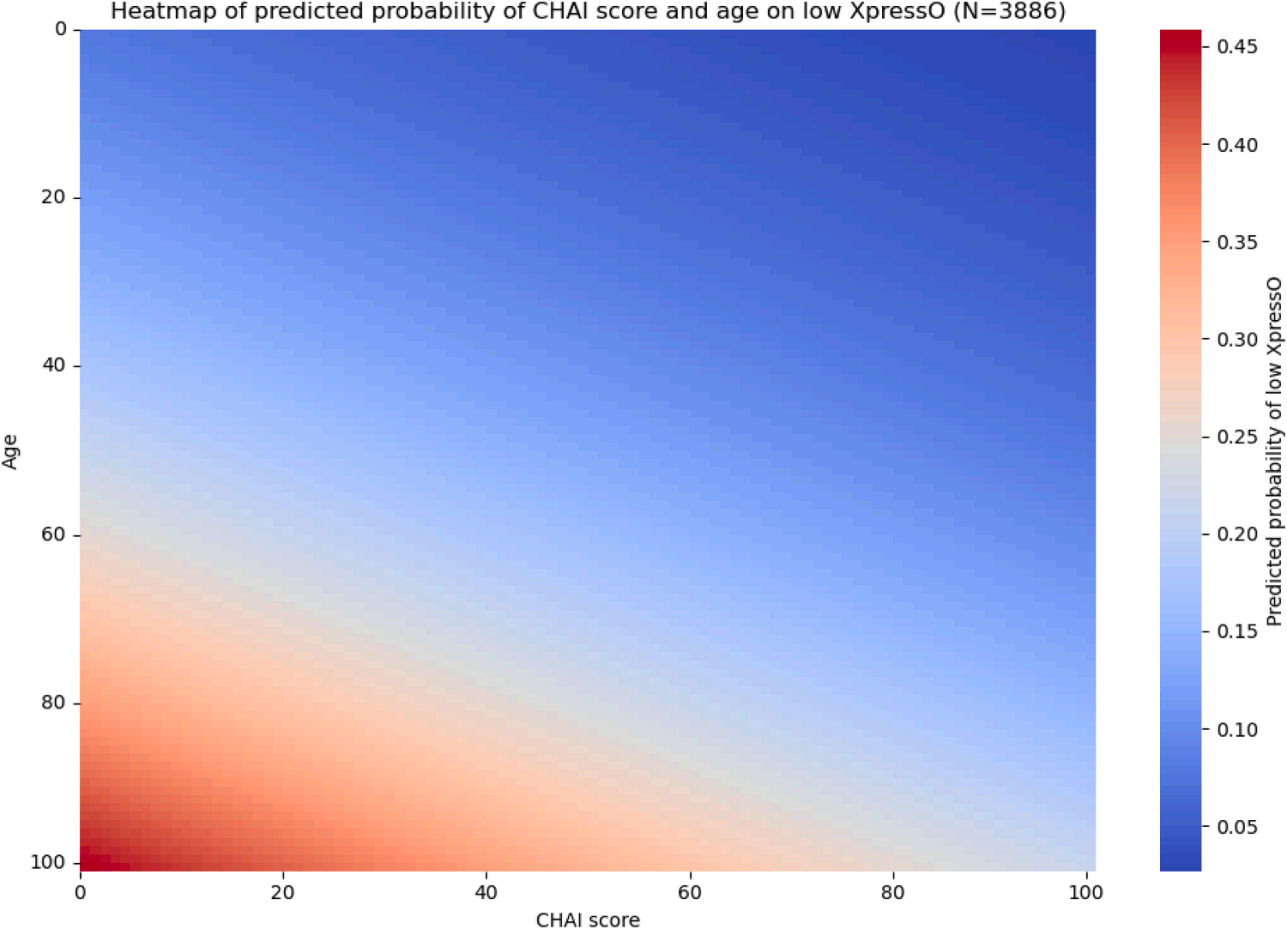
Heatmap of the predicted probability of MoCA-CHAI score and age on low XpressO score (<42).

## Appendix

The language used in the online MoCA-CHAI test is not a statistically significant predictor.

The language used in the online MoCA-CHAI test is not a statistically significant predictor, but gender is statistically significant, with higher odds for male persons, and with different age dependencies, as shown in Fig. A1.

The predicted probability of MoCA-CHAI score and age on low XpressO score (<42) is shown in Fig. A2. As can be seen, the predicted probability of a low XpressO score is higher with older age and lower CHAI score.

## Footnotes

1 For more information about the MoCA-CHAI see https://portal.mocacognition.com/cognitive-health
